# Agreement among Healthcare Professionals in Ten European Countries in Diagnosing Case-Vignettes of Surgical-Site Infections

**DOI:** 10.1371/journal.pone.0068618

**Published:** 2013-07-09

**Authors:** Gabriel Birgand, Didier Lepelletier, Gabriel Baron, Steve Barrett, Ann-Christin Breier, Cagri Buke, Ljiljana Markovic-Denic, Petra Gastmeier, Jan Kluytmans, Outi Lyytikainen, Elizabeth Sheridan, Emese Szilagyi, Evelina Tacconelli, Nicolas Troillet, Philippe Ravaud, Jean-Christophe Lucet

**Affiliations:** 1 Infection control unit, Bichat-Claude Bernard Hospital, Paris, France; 2 Bacteriology and Hygiene Department, Nantes University Hospital, Nantes, France; 3 Centre d’Épidémiologie Clinique, Hôpital Hôtel Dieu, Paris, France; 4 Medical microbiology and infection control, Southend University Hospital NHS Foundation Trust, Essex, United Kingdom; 5 Institute of Hygiene and Environmental Medicine, Charité, University Medicine Berlin, Berlin, Germany; 6 Department of Infectious Diseases and Clinical Microbiology, Ege University Medical Faculty, Bornova, Izmir, Turkey; 7 Institute of Epidemiology School of Medicine, Belgrade, Serbia; 8 Amphia Hospital Breda, Laboratory for Microbiology and Infection Control, Breda, the Netherlands; 9 Department of Infectious Disease, Epidemiology, National Public Health Institute, Helsinki, Finland; 10 Department of Healthcare-Associated Infection and Antimicrobial Resistance, HPA Centre for Infections, Colindale, London, United Kingdom; 11 Department of Epidemiology, Office of the Chief Medical Officer, Gyali, Hungary; 12 Department of Infectious Diseases, Università Cattolica del Sacro Cuore, Rome, Italy; 13 Division of Infectious Diseases, Central Institute of the Valais Hospitals, Sion, Switzerland; Universidad Nacional de La Plata., Argentina

## Abstract

**Objective:**

Although surgical-site infection (SSI) rates are advocated as a major evaluation criterion, the reproducibility of SSI diagnosis is unknown. We assessed agreement in diagnosing SSI among specialists involved in SSI surveillance in Europe.

**Methods:**

Twelve case-vignettes based on suspected SSI were submitted to 100 infection-control physicians (ICPs) and 86 surgeons in 10 European countries. Each participant scored eight randomly-assigned case-vignettes on a secure online relational database. The intra-class correlation coefficient (ICC) was used to assess agreement for SSI diagnosis on a 7-point Likert scale and the kappa coefficient to assess agreement for SSI depth on a three-point scale.

**Results:**

Intra-specialty agreement for SSI diagnosis ranged across countries and specialties from 0.00 (95%CI, 0.00–0.35) to 0.65 (0.45–0.82). Inter-specialty agreement varied from 0.04 (0.00–0.62) in to 0.55 (0.37–0.74) in Germany. For all countries pooled, intra-specialty agreement was poor for surgeons (0.24, 0.14–0.42) and good for ICPs (0.41, 0.28–0.61). Reading SSI definitions improved agreement among ICPs (0.57) but not surgeons (0.09). Intra-specialty agreement for SSI depth ranged across countries and specialties from 0.05 (0.00–0.10) to 0.50 (0.45–0.55) and was not improved by reading SSI definition.

**Conclusion:**

Among ICPs and surgeons evaluating case-vignettes of suspected SSI, considerable disagreement occurred regarding the diagnosis, with variations across specialties and countries.

## Introduction

Despite progress in prevention, [1] surgical-site infection (SSI) remains one of the most common adverse events in hospitals, accounting for 11% to 26% of all healthcare-associated infections [2]–[3]. SSI prevention is therefore receiving considerable attention from surgeons and infection control physicians (ICPs), healthcare authorities, the media, and the public in most European countries. There is a perception among the public that SSIs may reflect poor quality of care.

Several countries require public reporting of hospital-acquired infections, using either process indicators or infection rates, [4] with the goal of improving transparency, patient safety, public information, and performance by benchmarking of surgical units and healthcare facilities. However, there is little evidence that publishing quality indicators improves care [5]. The public reporting of infection rates remains debated at the national and international levels [6]. In any case, if SSI rates are to serve as a quality indicator for healthcare facilities and the public, they must be determined in a reliable way that produces robust infection rates [7].

SSI rates vary according to co-morbidities and to the contamination class and conditions of the surgical procedure. The need for adjustment has been demonstrated, and most surveillance networks use the National Nosocomial Infection Surveillance (NNIS) index for risk stratification [8]–[9]. Another factor that influences SSI rates is the robustness of SSI diagnosis. The extent to which different healthcare professionals agree about the presence of SSI depends on many factors including the use of a shared SSI definition, training, and experience. In several studies, the diagnosis of SSI varied according to the definitions used [10]. A recent French study documented considerable intra- and inter-specialty disagreement among healthcare professionals regarding the diagnosis of SSI [11]. Furthermore, recent studies from a European network suggested large differences in SSI recognition across countries [12].

We designed a study to assess agreement in SSI diagnosis among ICPs and surgeons involved in SSI surveillance in 10 European countries.

## Methods

### Ethics Committee Approval

Because of the observational and blinded nature of the study, the institutional review board of the Bichat-Claude Bernard Hospital waived the requirement for informed consent. According to this statement, written consents of patients were not collected. The study has been approved by the ethical committee of the Bichat-Claude Bernard Hospital group.

### Development of Case-vignettes

Case-vignettes allow an assessment of the same cases by ICPs and surgeons involved in diagnosing SSI. We used blinded random assignment of the case-vignettes to ICPs and surgeons to assess agreement regarding SSI diagnosis and depth. In addition, we determined whether providing SSI definitions influenced SSI diagnosis and depth assessment.

The case-vignettes were built from SSI surveillance data collected in six surgical units in four French university hospitals. Surgical procedures were selected based on the following criteria: i) preferentially clean or clean-contaminated surgical procedure; ii) presence of a skin incision allowing standard wound surveillance and SSI diagnosis, iii) surgical procedure usually requiring at least 1 week of in-hospital post-operative surveillance, and iv) sufficiently high SSI incidence to ensure the collection of a large number of suspected cases within a short period.

Consecutive patients with suspected SSI were followed throughout their hospitalisation or re-hospitalisation. Each day, a bedside evaluation was performed; the medical chart and nursing log were reviewed; and laboratory test results, microbiological findings, and imaging study findings were recorded. Photographs of the wound and/or computed tomography (CT) results were obtained. Suspected SSI was defined as wound modification or discharge and/or evidence of infection. We used the Centers for Disease Control SSI definition, which is identical to the European HELICS/IPSE definition [13].

We identified 20 patients with suspected SSI and complete information after heart surgery (n = 5), gastrointestinal surgery (n = 5), orthopaedic surgery (n = 4), obstetric surgery (n = 2), neurosurgery (n = 2), or ENT surgery (n = 2). A single investigator developed standardised case-vignettes, in English, based on these 20 patients. Each vignette described demographic data, past medical history, the surgical procedure, and the postoperative data. Figure S1 shows one of the case-vignettes.

### Participants

We asked 10 European leaders in SSI surveillance and prevention in 10 European countries (Finland, France, Germany, Hungary, Italy, Serbia, Switzerland, The Netherlands, Turkey, and the UK) to each recruit 10 ICPs and 10 surgeons for the study, using their personal connections, and to send the list of participants to the study investigators. Because of the observational and blinded nature of the study, the institutional review board of the Bichat-Claude Bernard Hospital waived the requirement for informed consent.

### Study Design and Data

The 20 vignettes were assigned at random to allow assessments of agreement among (i) participants in the same speciality in the same country; (ii) participants in the same speciality in different countries; and (iii) participants in different specialities in the same country.

Each of the 20 vignettes was to be scored four times by different ICPs and surgeons in all 10 countries. Then, four ICPs and four surgeons taken at random in each country read the SSI definitions and repeated the scoring of one vignette.

Scores were assigned using a seven-point Likert scale ranging from “SSI certainly absent” (score 1) to “SSI certainly present” (score 7) [14]. When the score was between 4 and 7, the participant scored SSI depth on a 3-point scale (1, superficial SSI; 2, depth unclear; and 3, deep or organ/space-related SSI). We simplified the depth assessment by classifying deep and organ/space-related SSIs in the same group, as both SSI categories have the same severe consequences in terms of mortality, morbidity, and hospital stay prolongation.

A secure online relational database was established for data collection. Each participant had a personal login and password [15]. Patient data were presented chronologically, and the scores assigned before reading the SSI definition could not be changed. Before scoring the vignettes, each participant provided the following information: age, gender, type of hospital, and time working in the current job.

### Statistical Analysis

We estimated the number of vignettes and participants needed to assess agreement within specialties based on the precision of the intra-class correlation coefficient (ICC) [16] and on feasibility considerations (number of participants available in each specialty, maximal time needed for scoring). With 20 vignettes each scored four times and an expected ICC of about 0.60, half the exact 95 per cent confidence interval (95%CI), i.e., precision, would be 0.29. Data were described as mean+/−SD, median (interquartile range), or percentage. Agreement was assessed before and after reading the SSI definition without distinguishing specialties or countries. To evaluate intra- and inter-specialty agreements for SSI diagnosis based on 1–7 Likert scale scores, we computed the ICC with the 95%CIs. An ICC of 0 indicates the level of agreement produced by chance alone and an ICC of 1 indicates perfect agreement. We defined poor agreement as ICC values less than 0.4, good agreement as ICC values of 0.4 to 0.7, and very good agreement as ICC values greater than 0.7 [17].

To evaluate intra- and inter-specialty agreement regarding SSI depth scored on a 3-point scale, we computed the kappa coefficient with the 95%CIs. We added a fourth category comprising the participants who did not score SSI depth because their SSI diagnosis score was less than 4. Agreement is considered poor when κ is 0.20 or less, fair when κ is 0.21–0.40, moderate when κ is 0.41–0.60, good when κ is 0.61–0.80, and very good when kappa coefficient is 0.81–1.00 [18]. Analyses were performed using SAS System, Version 9. 3 (SAS Institute, Cary, NC, USA).

## Results

### Characteristics of the Participants and Case-vignettes

Overall, 100 ICPs and 86 surgeons agreed to participate; there were 10 surgeons from each of six countries and four to nine surgeons from the remaining four countries. The 186 participants worked in publicly funded (n = 179) or private (n = 7) healthcare facilities in 75 university and 57 non-university hospitals; of these 132 hospitals, 95 (72%) each contributed one participant, 35 (27%) two or three participants, and two (1%) five participants. Median (IQR) age was 47 (40–53) years, 117 (62.9%) participants were men, median time in the current job was 13 (7–20) years, and 142 (76.3%) participants were directly involved in SSI surveillance programmes in their healthcare facility (Table S1).

Table S2 reports the characteristics of the 20 patients selected to build the case-vignettes. SSI was suspected before hospital discharge in 11 patients and after hospital discharge in 9 patients who required re-admission. Wound modification was a feature in 12 (60%) patients. Microbiological specimens were obtained from the surgical wound in 15 patients and were positive in 13.

As four countries contributed 14 fewer surgeons than expected, they contributed lower than expected scores. In all, each of the 20 vignettes was scored without the SSI definitions 40 times by ICPs; for scoring by surgeons, 8 vignettes were scored 35 times, 5 were scored 33 times, 3 were scored 34 times, and 4 had miscellaneous numbers of scorings. In all, there were 1488 scorings without the SSI definitions, instead of the expected 1600.

### Case-vignette Scores

In addition to the 1488 scorings without the SSI definitions, 14 vignettes were each scored four times and six vignettes three times with the SSI definitions, for a total of 74 scorings. The median SSI diagnosis score on the 7-point Likert scale obtained without reading the SSI definitions varied across countries from 6 to 7 for ICPs and from 5 to 7 for surgeons (Table 1).

**Table 1:** Distribution of scores assigned by infection control physicians and surgeons before reading the definitions of surgical site infections, on a 7-point Likert scale, in each of the ten European countries.

Infection Control Physicians				
Country	Number of vignettes scored	Mean (SD)	Median (IQR)	Min.–Max.
Turkey	80	5.2 (2.0)	6.0 (4.0–7.0)	1.0–7.0
Serbia	80	5.3 (2.3)	7.0 (3.0–7.0)	1.0–7.0
Hungary	80	5.2 (2.1)	6.0 (4.0–7.0)	1.0–7.0
Italy	80	5.3 (2.0)	6.0 (4.0–7.0)	1.0–7.0
Germany	80	5.4 (2.3)	7.0 (3.5–7.0)	1.0–7.0
Finland	80	5.0 (2.0)	6.0 (3.0–7.0)	1.0–7.0
The Netherlands	80	5.0 (2.1)	6.0 (3.0–7.0)	1.0–7.0
Switzerland	80	5.7 (1.9)	7.0 (4.5–7.0)	1.0–7.0
France	80	5.2 (2.0)	6.0 (3.0–7.0)	1.0–7.0
United Kingdom	80	5.3 (2.0)	6.0 (4.0–7.0)	1.0–7.0
**Surgons**				
Turkey	80	5.6 (2.0)	7.0 (4.0–7.0)	1.0–7.0
Serbia	80	5.0 (2.2)	6.0 (3.0–7.0)	1.0–7.0
Hungary	80	5.3 (2.1)	7.0 (3.0–7.0)	1.0–7.0
Italy	72	5.1 (2.2)	6.0 (3.0–7.0)	1.0–7.0
Germany	80	5.2 (2.1)	6.0 (3.0–7.0)	1.0–7.0
Finland	80	5.2 (2.0)	6.0 (3.0–7.0)	1.0–7.0
The Netherlands	32	5.4 (1.9)	6.0 (4.0–7.0)	2.0–7.0
Switzerland	80	6.0 (1.8)	7.0 (6.0–7.0)	1.0–7.0
France	72	4.5 (2.2)	5.0 (2.0–7.0)	1.0–7.0
United Kingdom	32	5.4 (1.8)	6.0 (4.5–7.0)	1.0–7.0
Total	688	5.2 (2.1)	6.0 (3.0–7.0)	1.0–7.0

SD, standard deviation; IQR, interquartile range; min, minimum; max, maximum.

### Intra-specialty and Inter-specialty Agreement Regarding SSI Diagnosis in each Country

For ICPs, the ICC based on scores assigned without reading the SSI definitions ranged across countries from 0.26 to 0.65. Agreement was best in Germany (ICC, 0.65; 95%CI, 0.45–0.82) and the UK (0.59, 0.38–0.80), good in four other countries, and poor in the four remaining countries. For surgeons, the ICC ranged across countries from 0.00 to 0.46. Agreement was good in Germany (ICC, 0.46; 95%CI, 0.23–0.69) and poor in the other nine countries (Table 2).

**Table 2:** Assessment of within- and between-specialty agreement about surgical site infection diagnosis within and across 10 European countries.

	SSI diagnosis score by ICPs, 7-point Likert scale (ICC)			SSI diagnosis score by surgeons, 7-point Likert scale (ICC)			SSI diagnosis score for all participants, 7-point Likert scale (ICC)				
	Number of vignettes scored	Without SSI definitions (95%CI)		Number of vignettes scored		Without SSI definitions (95%CI)		Number of vignettesscored		Without SSI definitions (95%CI)	
**Intracountry**											
Turkey	80	0.44 (0.22–0.68)	80		0.23 (0.03–0.50)			160	0.36 (0.20–0.58)		
Serbia	80	0.26 (0.05–0.53)	80		0.06 (0.00–0.35)			160	0.20 (0.04–0.41)		
Hungary	80	0.28 (0.07–0.54)	80		0.24 (0.04–0.51)			160	0.31 (0.16–0.54)		
Italy	80	0.31 (0.10–0.57)	72		0.08 (0.00–0.36)			152	0.20 (0.07–0.42)		
Germany	80	0.65 (0.45–0.82)	80		0.46 (0.23–0.69)			160	0.55 (0.37–0.74)		
Finland	80	0.30 (0.09–0.57)	80		0.21 (0.01–0.48)			160	0.30 (0.15–0.52)		
Netherlands	80	0.45 (0.23–0.69)	32		0.00 (0.00–0.35)			112	0.42 (0.23–0.65)		
Switzerland	80	0.40 (0.18–0.65)	80		0.08 (0.00–0.34)			160	0.26 (0.12–0.48)		
France	80	0.44 (0.22–0.68)	72		0.30 (0.07–0.57)			152	0.41 (0.24–0.63)		
United Kingdom	80	0.59 (0.38–0.80)	32		0.04 (0.00–0.62)			112	0.04 (0.00–0.62)		
**Intercountry**	800	0.41 (0.28–0.61)	688		0.24 (0.14–0.42)			1488	0.24 (0.14–0.42)		

SSI, surgical-site infection; ICP, infection control physician; ICC, intraclass correlation coefficient; 95%CI, 95% confidence interval.

The inter-specialty ICC based on scores assigned without reading the SSI definitions ranged across countries from 0.04 to 0.55. Agreement was best in Germany (ICC, 0.55; 95%CI, 0.37–0.74), good in two other countries and poor in the remaining seven countries (Table 2).

### Intra-specialty and Inter-specialty Agreement Regarding SSI Diagnosis Across Countries

The intra-specialty ICC was computed based on all scores in all countries. Agreement was good for ICPs (0.41, 0.28–0.61) and poor for surgeons (0.24, 0.14–0.42). Scoring after reading the SSI definitions improved agreement among ICPs (0.57, 0.20–0.82) but not among surgeons (0.09, 0.00–0.55). The inter-specialty ICC was estimated based on all 1488 scores obtained without reading the SSI definitions and showed poor agreement among the 186 participants (0.24, 0.14–0.42) (Table 2).

### Agreement Regarding SSI Depth

For ICPs, the intra-specialty kappa coefficient values for superficial/deep SSI scored without reading the SSI definitions varied across countries from 0.05 to 0.50 (Table 3). Agreement was moderate among German ICPs (0.50, 0.45–0.55) and fair (n = 8) or poor (n = 1) among ICPs in other countries. For surgeons, kappa coefficient varied from 0.05 and 0.31. Agreement was fair among German surgeons (0.31, 0.26–0.36) and lower in the other nine countries (Table 3). When all countries were pooled, agreement was fair among ICPs (0.28, 0.27–0.29) and poor among surgeons (0.19, 0.17–0.21) (Table 4).

**Table 3:** Assessment of within- and between-specialty agreement about surgical site infection depth within and across 10 European countries.

	SSI depth score by ICPs, 4-point scale^a^ (Kappa coefficient)				SSI depth score by surgeons, 4-point scale^a^ (Kappa coefficient)			
	Number of vignettes scored	Depth not scored(n, %)^b^	Without SSI definitions(95%CI)	Number of vignettes scored		Depth not scored(n, %)^b^		Without SSI definitions(95%CI)
**Intracountry**								
Turkey	80	19 (23.7)	0.26 (0.21–0.31)	80		17 (21.2)	0.25 (0.20–0.30)	
Serbia	80	21 (26.2)	0.05 (0.00–0.10)	80		25 (31.2)	0.05 (0.00–0.10)	
Hungary	80	18 (22.5)	0.20 (0.15–0.25)	80		22 (27.5)	0.16 (0.10–0.22)	
Italy	80	21 (26.2)	0.23 (0.17–0.29)	72		23 (31.9)	0.09 (0.01–0.17)	
Germany	80	20 (25.0)	0.50 (0.45–0.55)	80		21 (26.2)	0.31 (0.26–0.36)	
Finland	80	24 (30.0)	0.27 (0.22–0.32)	80		23 (28.7)	0.14 (0.09–0.19)	
The Netherlands	80	23 (28.7)	0.25 (0.20–0.30)	32		7 (21.9)	0.17 (0.00–0.34)	
Switzerland	80	11 (13.7)	0.34 (0.28–0.40)	80		18 (22.5)	0.14 (0.08–0.20)	
France	80	21 (26.2)	0.44 (0.39–0.49)	72		27 (37.5)	0.17 (0.09–0.25)	
United Kingdom	80	20 (25.0)	0.38 (0.32–0.44)	32		6 (18.7)	0.19 (−0.02–0.40)	
**Intercountry**	800	198 (24.7)	0.28 (0.27–0.29)	688		189 (27.5)	0.19 (0.17–0.21)	

SSI, surgical-site infection; ICP, infection control physician; 95%CI, 95% confidence interval.

a SSI Depth was scored on a 4-point scale: 1, no SSI in vignettes with scores lower than 4 on the 7-point Likert scale for SSI diagnosis; 2, superficial SSI; 3, uncertainty about SSI diagnosis; 4, deep/organ space SSI in vignettes scored 4 or more on the 7-point Likert scale for SSI diagnosis.

b SSI Depth not scored because infection scores were 3 or less.

**Table 4:** Overall assessment of agreement about surgical site infection depth within and across 10 European countries.

	SSI depth score by all participants, 4-point scale^a^ (Kappa coefficient)			
	Number of vignettes scored	Depth not scored (n, %)^b^	Without SSI definitions (95%CI)	
**Intracountry**				
Turkey	160	36 (22.5)	0.27 (0.24–0.30)	
Serbia	160	46 (28.7)	0.09 (0.07–0.11)	
Hungary	160	40 (25)	0.19 (0.16–0.22)	
Italy	152	44 (27.5)	0.31 (0.18–0.34)	
Germany	160	41 (25.6)	0.35 (0.32–0.38)	
Finland	160	47 (29.4)	0.25 (0.22–0.28)	
Netherlands	112	30 (18.7)	0.25 (0.18–0.32)	
Switzerland	160	29 (18.1)	0.24 (0.21–0.27)	
France	152	48 (30)	0.27 (0.23–0.31)	
United Kingdom	112	26 (16.2)	0.34 (0.25–0.42)	

a SSI Depth was scored on a 4-point scale: 1, no SSI in vignettes with scores lower than 4 on the 7-point Likert scale for SSI diagnosis; 2, superficial SSI; 3, uncertainty about SSI diagnosis; 4, deep/organ space SSI in vignettes scored 4 or more on the 7-point Likert scale for SSI diagnosis.

b SSI Depth not scored because infection scores were 3 or less.

The inter-speciality kappa coefficient for superficial/deep SSI scored without reading the SSI definitions varied across countries from 0.09 to 0.35, being highest in Germany. Reading the SSI definition did not change the scoring for superficial/deep SSIs by ICPs or surgeons (data not shown).

## Discussion

In a large panel of ICPs and surgeons involved in SSI surveillance in 10 European countries, agreement regarding the diagnosis and depth of SSI varied across countries and across individuals within both specialties. Reading the SSI definitions did not significantly improve agreement among ICPs or surgeons regarding the diagnosis or depth of SSI.

Although preventing all SSIs may not be feasible, bundles of peri- and postoperative measures have been developed to minimise the risk of SSI [9]. SSI surveillance is a component of these bundles. Reporting of SSI rates combined with active infection control efforts by qualified professionals and data feedback to the clinical staff has been shown to be a key factor in preventing SSIs [19]–[20]. At the local scale, incidence trends obtained by collecting SSI rates using same method over years provide information on the impact of preventive measures. At the global scale, SSI rates can theoretically be compared across units and hospitals. National SSI surveillance programmes have shown decreases in SSI rates, although the underlying mechanisms remain unclear [19], [21].

SSI surveillance is now strongly recommended and widely used in industrialised countries. National surveillance networks have issued standard surveillance protocols. However, SSI surveillance faces methodological challenges. If the SSI rate is to serve as a performance indicator, then valid and consistent SSI rates must be obtained. The challenge is both to obtain accurate information about the denominator characterising the study population and to accurately measure the number of SSIs (the numerator). Since 2008, the European Centre for Disease Prevention and Control (ECDC) has been monitoring SSI rates in Europe using a protocol established by the Hospitals in Europe Link for Infection Control through Surveillance (HELICS). A network of European countries that use the same surveillance methods was established, [22] and its results are published every year by the ECDC. However, the reliability and validity of infection reporting must be assessed regularly [23]. The main obstacles in interpreting SSI rates are variations in the diagnosis of SSI and in postoperative follow-up duration [24].

Several studies have documented imperfect agreement across physicians regarding the diagnosis of SSI. In one study, wide differences in SSI diagnosis were noted between ICPs and surgeons, as well as across surgeons [25]. In a recent study, surgeons tended to diagnose only deep and organ-space SSIs, whereas ICPs also diagnosed superficial SSIs, thereby doubling the total SSI rate [26]. A study comparing SSI rates from 11 European countries showed that the proportion of superficial SSIs varied from 20% to 80%, suggesting differences in SSI detection and/or classification across countries [12]. Finally, a study based on the same methodology as the one reported here assessed agreement among a large number of healthcare workers in France [11]. Agreement regarding SSI diagnosis and depth varied across specialties and across individuals within each specialty. Reading the SSI definition produced small improvements in agreement about SSI diagnosis and depth.

Our study further supports the existence of considerable uncertainty regarding SSI detection at the European level. Our results are probably reliable, as we placed the participants in unbiased conditions by asking them to score the same case-vignettes through an Internet database. This method ensured that the participants were not influenced by factors such as perceived SSI risk in a particular unit or patient. Considering such factors would probably have increased disagreement among participants. Disagreement may be higher regarding the diagnosis of post-discharge SSIs or of SSIs in patients with minimal wound discharge and no microbiological results.

We found scoring differences across participants, across countries, and across case-vignette types. Agreement for SSI diagnosis and depth was good in Germany within ICPs, within surgeons, and between both specialties. In Germany, the regular cross-hospital evaluations of diagnostic accuracy through case-vignettes, conducted as part of the KISS surveillance network, probably improve agreement [1]. Several other countries, such as The Netherlands, France, and the UK have had SSI surveillance networks for many years, which may have improved diagnostic accuracy via the sharing of surveillance methods and SSI rates. Providing the SSI definition did not improve the correlation between scores in our study, in keeping with a previous study demonstrating variable interpretations of the same definition [10]. Our results further support the need for a multidisciplinary approach to SSI surveillance [27]. Our data from 10 European countries consistently showed differences in agreement in each country, suggesting that our results may be also relevant to other countries.

Our study has several limitations. First, the vignettes were scored for the presence or absence of SSI by each participant working alone. SSI is often a difficult diagnosis that is typically made after discussion among surgeons and ICPs. Thus, SSI surveillance aims not only to obtain accurate SSI rates, but also to enhance teamwork between surgical and infection-control teams in order to ensure the implementation of effective preventive strategies. Our results indicate that surveillance should not be performed by individuals in a single specialty [27]. Second, the vignettes were scored via an online database. The vignettes were built from real cases, and the diagnosis of SSI may have been easier for surgeons or ICPs who had had direct contact with the patients. Third, the study was not designed to assess the accuracy of SSI diagnosis. Instead, we focused on agreement among ICPs and surgeons. We were therefore unable to determine which participants made the correct diagnosis, as illustrated in Table 1 showing SSI diagnosis score differences of up to 6 points between two participants from the same specialty. Fourth, we selected cases of suspected SSI to assess agreement about the diagnosis of SSI. However, SSI is suspected in only a small proportion of patients after surgery. Our data on agreement about SSI diagnosis would not apply to an actual series of surgical patients. Fifth, in some countries we did not reach the ten expected surgeons for participation. This lower than expected number of participants could have lead to a less precise analysis. Finally, participants in each country were contacted by European leaders in the field of SSI surveillance and prevention. This recruitment method may have lead to the selection of participants working in universities or high-level hospitals and, therefore, to overestimation of agreement in diagnosing SSI.

In conclusion, among ICPs and surgeons evaluating case-vignettes of possible SSI, considerable disagreement in SSI diagnosis occurred both between and within countries. This finding supports the need for caution when using SSI rates for benchmarking or public reporting. Nevertheless, SSI surveillance and feedback remain critical for SSI prevention, and must be encouraged despite intrinsic limitations. Rather than stopping SSI surveillance because of uncertain reliability, our results support regular evaluations of SSI diagnosis accuracy, with case-vignettes probably constituting a valuable educational tool.

## Supporting Information

Figure S1 **Example of a case-vignette developed for the study.**
(DOC)

Table S1 **Characteristics of the 186 study participants from 10 European countries.**
(DOC)

Table S2 **Characteristics of the 20 real patients used to develop the case-vignettes.**
(DOC)
