# Supplementary material for: Agreement among Healthcare Professionals in Ten European Countries in Diagnosing Case-Vignettes of Surgical-Site Infections
Source: PLoS One. 2013 Jul 9;8(7):e68618. doi: 10.1371/journal.pone.0068618 (PMC3706413; doi:10.1371/journal.pone.0068618)
Supplement: Figure S1 — Example of a case-vignette developed for the study. (DOC) [file pone.0068618.s001.doc]

**Figure S1:** Example of a case-vignette developed for the study

**Case #19: Orthopaedic surgery**

**Medical History**

- Age: 73 years - Weight: 113 Kg
- Past medical history: renal colic, hypertension
- Past surgical history: ocular cancer with implantation of a prosthesis in 1987, appendectomy at 18 years of age, total right hip replacement in 2008

**Hospital stay**

- Length of hospital stay: 29, December, 2010, to 10 January, 2011 =9 days
- Reason for admission: Total left hip replacement for osteoarthritis

**Surgical conditions**

- Surgery on 6 December 2010

- ASA score: 2 - Duration of surgery: 65 minutes

- Altemeier classification: 1 - Emergency surgery: no

- Endoscopic technique: no - Surgical implants: yes

- NNIS risk score: 0

**Prevention of post-operative infection**

- Skin preparation
  - - Pre-operative showering with skin antiseptics:
      - D-1: yes (povidone iodine soap)
      - D: yes (povidone iodine soap)
    - Surgical site disinfection: yes (povidone iodine + alcohol)
  - Prophylactic antibiotic: cefazolin, 2 g – IV - 30 minutes before the incision

**Surgical intervention**

- Surgical report dated 6 December 2010
  - General anaesthesia
  - Injuries observed: Damage to the hip joint
  - Surgical procedure: Total hip replacement with a short femoral shaft / drainage

**Post-operative course (1)**

- Follow-up in the surgical ward: no reoperation intended, afebrile patient, no pain to palpation, clean incision, no inflammation
- Favourable course - Hospital discharge on 13 December 2010 (postoperative day 7)

**Post-operative course (2)**

- Re admitted on postoperative day 23 for a discharge from the surgical incision

- Clinical examination: no fever, no pain to palpation, dehiscence of surgical incision with pus discharge and inflammation

**Post-operative course (3) Postoperative day 27**


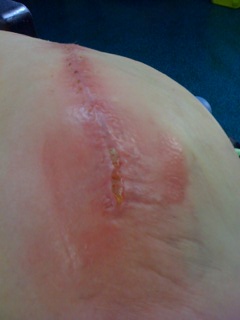

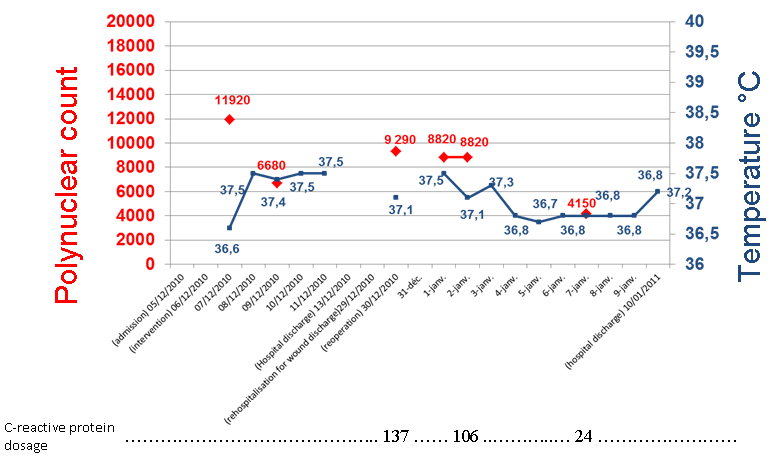


**Post-operative course (4)**

- Diagnosis and treatment: Empirical antibiotic therapy with vancomycin + gentamicin

- Needle aspiration of the wound on postoperative day 24

- Microscopy: multiple red cells, multiple leucocytes, rare cellular fragments, multiple Gram-positive cocci in clusters

- Culture: *Staphylococcus aureus,* fully susceptible except to penicillin

- Reoperation on postoperative day 28: incision and drainage of the subcutaneous abscess, aponeurosis preserved, culture of the drainage fluid was negative

- Favourable course - hospital discharge on 10 January 2011 (postoperative day 35)
