# Supplementary material for: Agreement among Healthcare Professionals in Ten European Countries in Diagnosing Case-Vignettes of Surgical-Site Infections
Source: PLoS One. 2013 Jul 9;8(7):e68618. doi: 10.1371/journal.pone.0068618 (PMC3706413; doi:10.1371/journal.pone.0068618)
Supplement: Table S1 — Characteristics of the 186 study participants from 10 European countries. (DOC) [file pone.0068618.s002.doc]

**Supplementary material:**

**Table S1.** Characteristics of the 186 study participants from 10 European countries

|  | **Turkey** | **Serbia** | **Hungary** | **Germany** | **Italy** | **Finland** | **The Nether-lands** | **Switzerland** | **France** | **UK** |
| --- | --- | --- | --- | --- | --- | --- | --- | --- | --- | --- |
|  | **N=20** | **N=20** | **N=20** | **N=20** | **N=19** | **N=20** | **N=14** | **N=20** | **N=19** | **N=14** |
| Age, median (IQR) | 48 (41-54) | 47 (41-54) | 47 (40-54) | 45.5 (40-54) | 46 (39-54) | 46 (40-54) | 45 (40-54) | 46 (40-54) | 44 (38-52.5) | 44 (38-52) |
| Age of ICPs (years, IQR)  Age of surgeons  Sex ratio (M/F) | 49.5 (47-54)  40 (37.5-51)  2.33 | 43 (42-49)  44 (41-56)  1.5 | 50 (47 -54)  42.5 (38-52)  1 | 47.5 (46-51)  43.5 (41-47)  0.6 | 47.5 (38-58)  48.5 (28-56)  1.22 | 48.5 (43-54)  52.5 (51-53)  0.81 | 41 (37-47)  47 (42-52)  1.8 | 54.5 (51-55)  47.5 (40-52)  5.67 | 44 (35-50)  34 (32-38)  3.75 | 45 (40-50)  44 (40-48)  1.33 |
| Number of HCFs  University/Public HCFs  Private facility  SSI surveillance  Time in current job, median | 13  13  0  13  10.5 (6-18) | 11  4  0  16  12 (6-20) | 12  6  0  12  13 (6-20) | 16  2  4  19  14 (6-21) | 14  8  1  10  13.5 (6-22) | 10  6  0  16  14.5 (7-24) | 12  10  0  11  15 (8-25) | 19  9  0  18  15 (10-25) | 15  11  1  17  14 (7.5-22) | 10  6  0  11  15 (8-23.5) |
|  |  |  |  |  |  |  |  |  |  |  |

ICP, infection-control physician; IQR, interquartile range; HCF, healthcare facility; SSI, surgical-site infection
