# Supplementary material for: Agreement among Healthcare Professionals in Ten European Countries in Diagnosing Case-Vignettes of Surgical-Site Infections
Source: PLoS One. 2013 Jul 9;8(7):e68618. doi: 10.1371/journal.pone.0068618 (PMC3706413; doi:10.1371/journal.pone.0068618)
Supplement: Table S2 — Characteristics of the 20 real patients used to develop the case-vignettes. (DOC) [file pone.0068618.s003.doc]

**Table S2.** Characteristics of the 20 real patients used to develop the case-vignettes

|  | **Heart surgery** | **Gastrointes-tinal surgery** | **Orthopaedic**  **surgery** | **ENT**  **surgery** | **Obstetrical**  **surgery** | **Neuro**  **surgery** |
| --- | --- | --- | --- | --- | --- | --- |
|  | **N=5** | **N=5** | **N=4** | **N=2** | **N=2** | **N=2** |
| Age in years, median (IQR) | 65 (61-76) | 62 (61-63) | 68.5 (64-74) | 58.5 (56-61) | 28.5 (27-30) | 61 (55-68) |
| Females, n (%) | 3 (60) | 2 (40) | 3 (75) | 1 (50) | 2 (100) | 1 (50) |
| Body mass index, median (IQR) | 31 (29-33) | 26 (24-31) | NA | NA | NA | 32.5 (28.7-36.2) |
| Diabetes mellitus, n (%) | 1 (20) | 1 (20) | 1 (25) | 1 (50) | 0 (0) | 1 (50) |
| ASA score ≥ 3, n (%) | 5 (100) | 0 (0) | 0 (0) | 0 (0) | 0 (0) | 2 (100) |
| Operative time in minutes, median (IQR) | 255 (230-270) | 260 (130-270) | 92.5 (62-121) | 270 (255-285) | 245 (143-348) | 270 (165-375) |
| Laparoscopic procedure, n (%) | 0 (0) | 2 (40) | 0 (0) | 0 (0) | 0 (0) | 0 (0) |
| Prophylactic antibiotics, n (%) | 5 (100) | 5 (100) | 4 (100) | 2 (100) | 2 (100) | 2 (100) |
| Days from surgery to SSI suspicion, median (IQR) | 12 (6-14) | 6 (4-8) | 10.5 (9.7-14) | 10 (6.5-13.5) | 9 (8-10) | 27 (21.5-32.5) |
| Wound modification, n (%) | 4 (80) | 3 (60) | 1 (25) | 2 (100) | 1 (50) | 1 (50) |
| Leucocytes >10 000/mm^3^, n (%) | 4 (80) | 4 (80) | 1 (25) | 1 (50) | 1 (50) | 1 (50) |
| Fever >38°C, n (%) | 2 (40) | 3 (60) | 0 (0) | 0 (0) | 2 (100) | 1 (50) |
| Microbiological wound sample, n (%) | 5 (100) | 1 (20) | 3 (75) | 2 (100) | 2 (100) | 2 (100) |
| If yes, |  |  |  |  |  |  |
| Neutrophils upon microscopy, n (%) | 5 (100) | 5 (100) | 1 (25) | 2 (100) | 2 (100) | 2 (100) |
| Significant microorganism, n (%) | 3 (60) | 5 (100) | 4 (100) | 2 (100) | 2 (100) | 2 (100) |
| Antibiotic treatment, n (%) | 3 (60) | 4 (80) | 3 (75) | 2 (100) | 2 (100) | 2 (100) |
| Re-operation, n (%) | 2 (40) | 1 (20) | 2 (50) | 2 (100) | 1 (50) | 2 (100) |

IQR, interquartile range; ASA, American Society of Anesthesiology (the ASA score include five classes, with higher score denoting higher risk of anaesthesia complications and surgical-site infection); NA, Non available
